# Postsurgery Classification of Best-Corrected Visual Acuity Changes Based on Pterygium Characteristics Using the Machine Learning Technique

**DOI:** 10.1155/2021/6211006

**Published:** 2021-11-15

**Authors:** Fatin Nabihah Jais, Mohd Zulfaezal Che Azemin, Mohd Radzi Hilmi, Mohd Izzuddin Mohd Tamrin, Khairidzan Mohd Kamal

**Affiliations:** ^1^Kulliyyah of Allied Health Sciences, International Islamic University Malaysia, Bandar Indera Mahkota, Kuantan 25200, Pahang, Malaysia; ^2^Kulliyyah of ICT, International Islamic University Malaysia, Gombak, Kuala Lumpur 50728, Malaysia; ^3^Kulliyyah of Medicine, International Islamic University Malaysia, Bandar Indera Mahkota, Kuantan 25200, Pahang, Malaysia

## Abstract

**Introduction:**

Early detection of visual symptoms in pterygium patients is crucial as the progression of the disease can cause visual disruption and contribute to visual impairment. Best-corrected visual acuity (BCVA) and corneal astigmatism influence the degree of visual impairment due to direct invasion of fibrovascular tissue into the cornea. However, there were different characteristics of pterygium used to evaluate the severity of visual impairment, including fleshiness, size, length, and redness. The innovation of machine learning technology in visual science may contribute to developing a highly accurate predictive analytics model of BCVA outcomes in postsurgery pterygium patients.

**Aim:**

To produce an accurate model of BCVA changes of postpterygium surgery according to its morphological characteristics by using the machine learning technique. *Methodology*. A retrospective of the secondary dataset of 93 samples of pterygium patients with different pterygium attributes was used and imported into four different machine learning algorithms in RapidMiner software to predict the improvement of BCVA after pterygium surgery.

**Results:**

The performance of four machine learning techniques were evaluated, and it showed the support vector machine (SVM) model had the highest average accuracy (94.44% ± 5.86%), specificity (100%), and sensitivity (92.14% ± 8.33%).

**Conclusion:**

Machine learning algorithms can produce a highly accurate postsurgery classification model of BCVA changes using pterygium characteristics.

## 1. Introduction

Pterygium is an ocular pathology where a form of triangular or wing-shaped fibrovascular connective tissue grows from the limbus and covers the corneal surface [1]. The interference of the tissue towards the cornea leads to the visual disruption that causes irritation, inflammation, tearing, dryness, and itchiness. The etiology of pterygium is still unknown; however, excessive ultraviolet (UV) light exposure due to hot climates or spending lots of time in a humid sunny environment is the prominent factor that contributes to this condition [2]. Pterygium can be categorized as inactive or active depending on its progression over time, developing corneal distortion when the active form occurs [1]. The main concern is a significant refractive change that eventually results in visual impairment if it remains untreated for a long time. Refractive change due to pterygium is characterized as corneal astigmatism due to flattening of the cornea, and it is treatable since the affected vision from the active growth of the conjunctiva tissue can be managed accordingly with surgical intervention [1].

Best-corrected visual acuity (BCVA) and contrast sensitivity function are the standard parameters for quantifying visual impairment due to corneal changes in the pterygium [3]. BCVA is defined as measuring the optimum visual function representing the best vision with the best corrective lenses by using a standard eye chart, Snellen, or logarithm minimum angle of resolution (LogMAR). Different morphologies of pterygium can be classified into three levels as an indicator of its severity according to its clinical appearance, which identified as atrophy (grade 1), intermediate (grade 2), and fleshy (grade 3) [4]. The grading was determined based on the presentation of the fleshiness or episcleral vessel under the body of pterygium relative to the transparency loss of fibrovascular tissue of pterygium. Some researchers focused on the size and length of pterygium as an indication of pterygium surgery. However, the severity of visual impairment did not correlate with the two determinants, and the results vary from one author to another [3]. The contradiction between the studies shows that there is a lack of agreement on specific determinants of pterygium progression that can cause ineffective management, especially for early surgical intervention of pterygium patients. Therefore, machine learning technology may be an excellent strategy to generate an accurate prognosis of visual impairment in pterygium patients.

Machine learning application assists in the prediction of future outputs by learning the information from the previous input in the system. It has been described extensively in several studies that have the potential to produce high accuracy of a classification model [5]. This approach has been beneficial for many medical practices, including diabetes [6], heart disease [5], and in clinical vision sciences [7]. In the same way, it may be applied in the pterygium-based study to assess the visual performance of BCVA changes according to its morphology upon pterygium surgery. This study aims to discover the best machine learning algorithms that can produce an accurate predictive analytics model to identify the most significant determinants of BCVA changes.

Alternatively, ensembled methods provide the promising mechanism of applying multiple iteration of one or combination of different machine learning algorithms but with a different subset of the training dataset as the input used for every iteration. The results are aggregated and expected to yield a higher prediction accuracy than merely the machine learning algorithms as the base learner. The most popular ensembled methods are bagging, AdaBoost, and majority vote. They can minimize overfitting that often create inconsistent prediction with the manifestation of the variance in the machine learning algorithms. There are many prominent applications that employed the ensembled methods in text classification such as the sentiment classification [8], genre classification [9], web page classification [10], and biomedical data classification [11].

## 2. Methods

### 2.1. Study Design and Data Acquisition

This retrospective data analysis study used a secondary dataset of 93 samples of pterygium patients from the previous study of Hilmi et al. [3], with an outcome of BCVA magnitude (logMAR). The magnitude of BCVA was calculated by the difference between postsurgery and presurgery BCVA. The six attributes of the quantitative data included in this study were Donald's pterygium grading (grade 1, grade 2, grade 3, and grade 4) of Tan et al. [4], fibrovascular redness (scales 1–3), thickness (mm), length (mm), total area (px), and dry weight (mcg).

### 2.2. Machine Learning Algorithms and Data Processing

The imported data consisted of six attributes and category labels of the BCVA outcome. The attributes of redness, thickness, length, total area, and dry weight were categorized as a real number. Meanwhile, the attribute of pterygium grading and label of BCVA was selected as a binomial. The prediction of the outcome of BCVA changes was discrete in the category label of “YES” as improved or “NO” as not improved. The outcome was considered to improve when the magnitude of BCVA was more than zero (>0), while the outcome of zero (=0) implies no improvement. Next, the data were split into the training and testing phases to develop and evaluate the model for each algorithm.

A predictive analytics software of RapidMiner Studio Version 9.8. was used to conduct a machine learning analysis with four machine learning algorithms: decision tree, support vector machine (SVM), logistic regression, and Naïve Bayes. The decision tree is in the form of a tree from the breakdown of the input dataset to smaller subsets from one category. The tree starts from the root to leaf nodes, while each leaf node is assigned a class label. This algorithm requires less learning time and simple to manage the missing values. The best split point is chosen based on the one that can trigger the smallest reduction in the impurity. The entropy measure (or the impurity measure) for the split point of *A*, the area of division, is defined as follows:

(1) $$\begin{array}{c} \text{Entropy}\left(A\right)=-\sum_{k=1}^{2}p_{k}\log_{2}\left(p_{k}\right), \end{array}$$

where *p*_*k*_ refers to the proportion of the records in category label *k* of the BCVA outcome. The value ranges from 0 to log_2_(2) in that 0 indicate the whole records belong to only one category label, whereas log_2_(2) indicates that the two category labels of the BCVA outcome are represented equally. Meanwhile, SVM outputs an optimal hyperplane that separates the input dataset into classes. A linear classification and division process are executed for selecting a line that has the highest margin of safety to perform the SVM classifier. The hyperplane of *x*, the input dataset, is defined as follows:

(2) $$\begin{array}{c} h\left(x\right)=\text{sign}\left(w^{T}x+b\right), \end{array}$$

where *w* is the vector, *b* is the biased term, and *x* is the input dataset. On the other hand, logistic regression can classify a categorical dependent variable or a binary outcome based on independent variables, either discrete or continuous data. The logistic regression is modelled as a nonlinear function of its predictor attributes as follows:

(3) $$\begin{array}{c} p=\frac{1}{1+\;e^{-\left(\beta_{o}+\;\beta_{1}x_{1}+\;\beta_{2}x_{2}+\cdots +\;\beta_{6}x_{6}\right)}}, \end{array}$$

where *p* is the probability of belonging to the category labels of the BCVA outcome. The variables *x*_1_, *x*_2_,…, *x*_6_ represent the six predictor attributes and their coefficients *β*_1_, *β*_2_,…, *β*_6_, respectively. In contrast to the Naïve Bayes classifier, the probability of each feature or characteristic was treated independently and did not depend on other features. It is a probabilistic approach that is based on the Bayes theorem. The Bayes classifier is defined as follows:

(4) $$\begin{array}{c} P\left(C_{1}|x_{1},x_{2},\ldots ,x_{6}\right)=\frac{P\left(C_{1}\right)\left[P\left(x_{1}|C_{1}\right)P\left(x_{2}|C_{1}\right)\cdots P\left(x_{6}|C_{1}\right)\right]}{P\left(C_{1}\right)\left[P\left(x_{1}|C_{1}\right)P\left(x_{2}|C_{1}\right)\cdots P\left(x_{6}|C_{1}\right)\right]+P\left(C_{2}\right)\left[P\left(x_{1}|C_{2}\right)P\left(x_{2}|C_{2}\right)\cdots P\left(x_{6}|C_{2}\right)\right]}, \end{array}$$

where *P* is the probability with the given set of six predictor attribute values, *x*_1_, *x*_2_,…, *x*_6_ belongs to the first category labels of the BCVA outcome, and *C*_1_ and *C*_2_ denote both prediction classes of BCVA changes.

The performance of the individual machine learning techniques might be improved by combining base learner algorithms through ensemble methods as evident in the previous works [11]. For comparison with base learner classifiers, common ensemble algorithms were employed in this study including majority voting, AdaBoost, and bagging. Only the best base learner was selected for AdaBoost and Bagging implementation.

Next, the data were split into a training and testing phases for development and evaluation of the model for each algorithm. A 10-fold cross-validation strategy was used to avoid bias in data partitioning. The dataset was divided into ten partitions (90% training and 10% testing), and the average results were used to estimate the model accuracy.

### 2.3. Classification Performance Measurements

The performance of the classification model was evaluated by its accuracy, sensitivity, specificity, and precision. The analysis of performance was expressed in true positive (TP), true negative (TN), false positive (FP), and false negative (FN). The measurements of each performance parameter were calculated as follows:

(5) $$\begin{array}{c} \text{Accuracy}=\frac{\left(\text{TP}+\text{TN}\right)}{\left(\text{TP}+\text{TN}+\text{FP}+\text{FN}\right)},\\ \text{Sensitivity}=\frac{\text{TP}}{\left(\text{TP}+\text{FN}\right)},\\ \text{Specificity}=\frac{\text{TN}}{\left(\text{TN}+\text{FP}\right)},\\ \text{Precision}=\frac{\text{TP}}{\left(\text{TP}+\text{FP}\right)}. \end{array}$$

**Figure 1:**
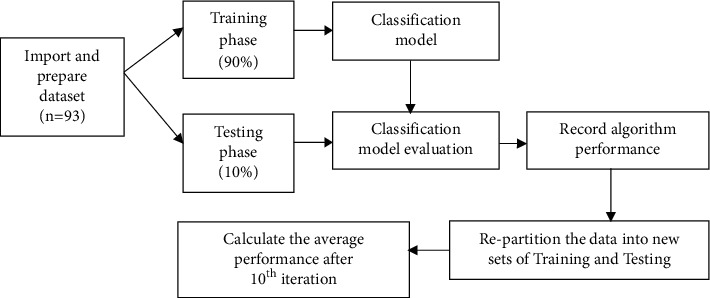
Flowchart of supervised machine learning with 10-fold cross-validation.

The area under the ROC (receiver operating characteristic) curve (AUC), which represents the area of sensitivity versus specificity, was computed to compare the binary classification model with one and another. The higher AUC value displays a better model's ability to correctly classify the positive and negative classes from a random sample. The overview of the supervised machine learning analysis with 10-fold cross-validation is shown in Figure 1.

## 3. Results

### 3.1. Decision Tree

**Figure 2:**
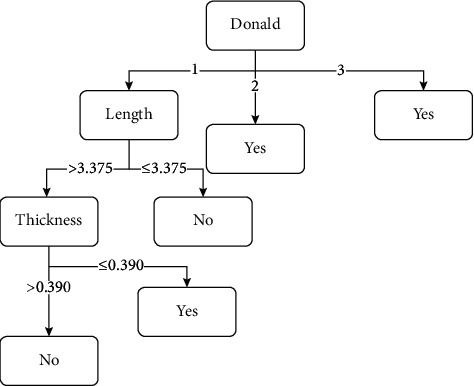
Decision tree model.

The decision tree model as shown in Figure 2 presents that the most significant attribute that contributes to the improvement of BCVA after pterygium surgery was Donald's pterygium grading by Tan et al. [4]. BCVA was likely to improve for pterygium patients with grades 2 and 3. The patients with grade 1 and less than 3.375 mm length of pterygium were predicted to have no improvement of BCVA. On the other hand, patients with grade 1 and the length of pterygium more than 3.375 mm were required to refer to its thickness. The thickness that was 0.39 mm or less predicted the BCVA would improve and vice versa.

### 3.2. Support Vector Machine

**Figure 3:**
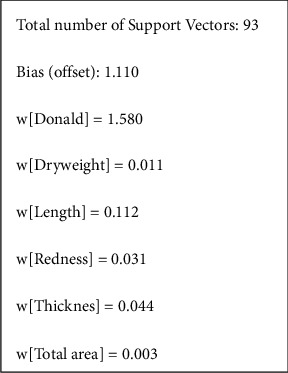
Support vector machine (SVM) model.

The SVM model displays the weight of each attribute that influences the improvement of BCVA based on the weighted kernel function. From Figure 3, the highest value was predominated by Donald's grading of pterygium (1.580), which signifies the main attribute to the BCVA improvement, followed by length (0.112), thickness (0.044), redness (0.031), dry weight (0.011), and total area (0.003).

### 3.3. Logistic Regression

The logistic regression model in Table 1 presents each attribute with a regression coefficient which denotes the positive or negative correlation between the attributes and the outcome of BCVA improvement. The highest coefficient in the regression coefficient was 456.434 (*p* ≤ 0.001) with Donald's grading, representing a positive coefficient of maximum likelihood to produce an outcome of BCVA improvement after pterygium surgery. The second attribute was the length (124.609, *p* ≤ 0.001), followed by redness (49.445, *p*=0.037) and total area (0.012, *p*=0.272). On the other hand, dry weight and thickness were negatively correlated with −0.436 (*p*=0.099) and −1343.404 (*p*=0.003), respectively.

### 3.4. Naïve Bayes

The model of probability function of BCVA improvement was in the form of Gaussian that follows a normal distribution with the means and standard deviations given in Table 2. The center of the graph is determined by the mean of the distribution, while the standard deviation determines the height and width of the graph. The overlapping area by the separation between the two graphs displays the ability of the attributes to discriminate between two outcomes. It was found that the overlapping area in redness was higher than other attributes that depict redness has lower probability of measuring the difference in BCVA outcomes. The blue graph of Donald's grading was too narrow due to low standard deviation (0.001); this indicates that the data points were clustered very close to the mean and show better reliability.

### 3.5. Classification Model Performance

As given in Table 3, the postpterygium surgery classification of the BCVA base learner model that achieves the highest accuracy was SVM (94.44% ± 5.86%), followed by decision tree (95.56% ± 5.74%), Naïve Bayes (94.44% ± 7.86%), and logistic regression (91.22% ± 8.74%). The SVM model's specificity and precision were the highest at 100% compared with the other base learner models. On the contrary, the SVM model had the lowest percentage of sensitivity (92.14% ± 8.33%) as the decision tree model reached the highest sensitivity (96.67% ± 7.03%). Nevertheless, the performance of the base learner SVM model was represented by the AUC value displaying the best prediction's quality with 0.983 ± 0.053. The AUC rank was followed by Naïve Bayes, logistic regression, and finally was the decision tree. Ensemble methods did not significantly increase the overall classification performance in the current dataset.

## 4. Discussion

The characteristics of pterygium tissue can be interpreted by its fleshiness, redness, dry weight, thickness, length, and total area. However, the application of each feature in clinical decision making and predicting the outcome of postpterygium surgery is subjective and inconsistent. In this study, the classification of prediction of best-corrected visual acuity (BCVA) outcome is essential in the prognosis as well as the indicator plan of pterygium excision surgery. The classification model of different supervised machine learning algorithms in RapidMiner demonstrated that the support vector machine (SVM) outperformed other classifiers in overall performance. Even so, all algorithms' overall performance presented high accuracy, sensitivity, and specificity, although there is a great variance in their concept. SVM is a commonly used algorithm used in clinical vision sciences, including in the diagnosis of age-related macular degeneration and diabetic retinopathy [7]. This algorithm aims to create the best boundary of a hyperplane to separate between different classes in a dataset by maximizing the width between the closest points. The interpretability of SVM was in the form of the weight of each attribute, which the highest positive weight likely indicates a significant feature. Nonetheless, in comparison with decision tree algorithms, it is more straightforward to comprehend through a complete tree model structure with attributes, value, and rules, especially when applied in clinical practice. Although machine learning has been popularly employed in the healthcare system, it is challenging to be implemented as it is indicated as a “black box,” following the absence of explanation underlying the input and output of data.

The SVM model presented Donald's pterygium grading based on its fleshiness significantly associated with the improvement of BCVA after surgery. This clinical grading of the pterygium's fleshiness was proposed by Tan et al. [4] by classifying it into atrophy, intermediate or fleshy relative to the transparency loss that corresponds to the increased fleshiness of the fibrovascular pterygium tissue that can be observed through the slit lamp. Atrophy (grade 1) was described with prominent and clear episcleral vessels. Meanwhile, grade 3 (fleshy) was classified by the total indistinct of an episcleral vessel under the body of pterygium. On the contrary, the appearance of episcleral that was labelled as partial, indistinct, or vague could fall in between the two categories as in grade 2 (intermediate). To the best of our knowledge, there is no research study yet that discusses BCVA changes of postpterygium surgery by clinical morphology of pterygium's fleshiness. Comparatively, Sandra et al. [12] reported the fleshiness-influenced pterygium recurrence after conjunctival autografting surgery with fibrin glue. Another study by Khan and Niazi [13] demonstrated that the recurrence rate difference between the fleshy and atrophic pterygium groups was statistically significant (*p*=0.01) among the three groups. The recurrence rate of pterygium was 9% (5 out of 55 eyes), 18% (10 out of 55 eyes), and 27.3% (15 out of 55 eyes) for the atrophic, intermediate, and fleshy groups, respectively. Other morphology characteristic such as length was statistically insignificant with the pterygium recurrence rate [13]. However, it was contradicted by Dzunic et al. [14] and Ha et al. [15] in which they presented the postoperative proliferation that induces the recurrence rate was not associated with the morphology. The contradiction could be due to different surgical techniques as the former used the bare sclera method covered with direct suture of the conjunctival wound, meanwhile the latter involved amniotic membrane transplant.

On the other hand, a few studies have researched evaluating the effect of pterygium excision in BCVA but with different morphologic features. Instead of pterygium's fleshiness, the grade was based on the extension of the apex of the pterygium of the cornea with four grades [16, 17]. It was found that the BCVA improvement was statistically significant in grades II, III, and IV, in which the pterygium was described as crossing the limbus of the cornea between 2 mm and 4 mm or more than 4 mm and reaching up to the pupillary margin or had crossed the pupillary margin. Conversely, grade I, with the pterygium crossing the limbus by less than 2 mm from the cornea, was not significant with the improvement of BCVA (*p*=0.184) [17]. According to Razmjoo et al. [18], the reorganization of the surface of the tear film resulting from the mechanical effects induced by pterygium leads to the improvement of BCVA after excision surgery. Despite the different morphology features from our findings, the idea of standardizing the clinical grading of pterygium certainly could contribute to the prediction of the refractive outcome of the surgery.

While previous works have shown promising results through ensemble methods, the current dataset did not indicate any significant increase in performance. Models developed using base learners also have the advantage of interpretability in terms of the rule formulated (e.g., decision tree) and weights associated with the attributes (e.g., SVM).

This study's limitation is the small amount of data used, and we observed that small alterations in the dataset could influence the prediction model. Overall, the development of machine learning in healthcare plays a fundamental role in assisting proper management and diagnosis. It can also be applied in finding a surgical indicator as well as prevention of recurrence. Additionally, this innovation allows healthcare practitioners to develop skills and knowledge of the concept of machine learning and artificial intelligence.

## 5. Conclusion

In summary, machine learning algorithms can produce a postsurgery classification model of BCVA changes using pterygium characteristics with high accuracy, specificity, and sensitivity. The best-performing algorithm in this study is the support vector machine (SVM), which outperforms other algorithms including decision tree, logistic regression, and Naïve Bayes. The SVM model exhibited that pterygium characteristic of Donald's grading by its fleshiness was the highest feature weight in association with BCVA changes in postpterygium surgery. It is suggested to perform further research on specific attributes of fleshiness following Donald's grading by Tan et al. [4] in affecting BCVA changes of postpterygium surgery by using the machine learning technique.

## Figures and Tables

**Table 1:** Logistic regression model.

Attribute	Coefficient	Standard coefficient	Standard error	*z* value	*P* value
Donald	456.434	371.630	87.931	5.191	≤0.001
Redness	49.445	27.240	23.664	2.089	0.037
Thickness	−1343.404	−149.799	448.471	−2.996	0.003
Length	124.609	217.007	29.668	4.200	≤0.001
Total area	0.012	13.328	0.011	1.099	0.272
Dry weight	−0.436	−32.601	0.264	−1.651	0.099
Intercept	−420.476	350.923	133.025	−3.161	0.002

**Table 2:** Naïve Bayes model.

Attribute	Parameter	No	Yes
Total area	Mean	1392.000	1999.552
	Standard deviation	778.824	1189.886
Donald	Mean	1.000	2.382
	Standard deviation	0.001	0.624
Dry weight	Mean	130.717	211.152
	Standard deviation	48.460	71.205
Length	Mean	2.314	3.887
	Standard deviation	0.836	1.800
Redness	Mean	1.648	2.130
	Standard deviation	0.539	0.499
Thickness	Mean	0.372	0.491
	Standard deviation	0.065	0.108

**Table 3:** Performance of classification models by cross-validation.

	Accuracy (%)	Specificity (%)	Sensitivity (%)	Precision (%)	AUC
Support vector machine (SVM)	94.44 ± 5.86	100.00	92.14 ± 8.33	100.00	0.983 ± 0.053
Decision tree	95.56 ± 5.74	91.67 ± 18.00	96.67 ± 7.03	97.32 ± 5.66	0.550 ± 0.158
Naïve Bayes	94.44 ± 7.86	96.67 ± 10.54	93.57 ± 8.33	98.33 ± 5.27	0.967 ± 0.070
Logistic regression	91.22 ± 8.74	83.33 ± 32.39	93.57 ± 8.33	94.86 ± 8.59	0.961 ± 0.123
Ensemble vote	95.56 ± 5.74	100.00	93.57 ± 8.33	100.00	0.792 ± 0.252
Ensemble AdaBoost-SVM	94.44 ± 10.8	93.33 ± 21.08	95.24 ± 7.69	97.14 ± 9.04	0.986 ± 0.044
Ensemble bagging-SVM	94.44 ± 5.86	100.00	92.14 ± 8.33	100.00	0.983 ± 0.053

## Data Availability

The machine learning data used to support the findings of this study are restricted by the IIUM Ethics Committee in order to protect patient privacy. Data are available from Mohd Radzi Hilmi, mohdradzihilmi@iium.edu.my, for researchers who meet the criteria for access to confidential data.
